# Hope on the horizon: Wharton's jelly mesenchymal stem cells in the fight against COVID-19

**DOI:** 10.2217/rme-2023-0077

**Published:** 2023-08-09

**Authors:** Gaurav Gupta, Md Sadique Hussain, Riya Thapa, Rajiv Dahiya, Debarshi Kar Mahapatra, Asif Ahmad Bhat, Neelam Singla, Vetriselvan Subramaniyan, Sushama Rawat, Vikas Jakhmola, Roshan S, Kamal Dua

**Affiliations:** ^1^School of Pharmacy, Suresh Gyan Vihar University, Jagatpura, Mahal Road, Jaipur, India; ^2^Center for Global Health research (CGHR), Saveetha Institute of Medical & Technical Science (SIMATS), Saveetha University, Chennai, India; ^3^School of Pharmaceutical Sciences, Jaipur National University, Jagatpura, Jaipur, Rajasthan, India; ^4^School of Pharmacy, Faculty of Medical Sciences, The University of the West Indies, Saint Augustine, Trinidad & Tobago; ^ 5^Department of Pharmaceutical Chemistry, Dr. D. Y. Patil Institute of Pharmaceutical Sciences and Research, Pimpri, Pune 411018, Maharashtra, India; ^6^Department of Pharmacology, Jeffrey Cheah School of Medicine & Health Sciences, MONASH University, Malaysia; ^7^Uttaranchal Institute of Pharmaceutical Sciences, Uttaranchal University, Dehradun, India; ^8^Deccan School of Pharmacy, Hyderabad, India; ^9^Faculty of Health, Australian Research Centre in Complementary & Integrative Medicine, University of Technology, Sydney, Ultimo-NSW 2007, Australia; ^10^Discipline of Pharmacy, Graduate School of Health, University of Technology, Sydney, Ultimo-NSW 2007, Australia

**Keywords:** COVID-19, mesenchymal stem cells, tissue regeneration

With millions of people infected and hundreds of thousands of fatalities worldwide, the global health and economies have been greatly affected by the COVID-19 pandemic. The respiratory system is the main target of SARS-CoV-2, a virus that can lead to serious pneumonia and even ARDS, a condition that can be fatal. The absence of a particular cure for COVID-19 has made it a worldwide priority to create effective therapies. One potential therapeutic approach is the use of Wharton's jelly mesenchymal stem cells (WJ-MSCs) [1]. WJ-MSCs are stem cells derived from the umbilical cord. Specifically, they are obtained from Wharton's jelly, which is a gelatinous substance that surrounds and sustains the blood vessels within the cord. WJ-MSCs can differentiate into various cell types, including adipose, cartilage, bone as well as cells of the nervous system like neurons and glial cells [2]. Additionally, they possess the ability to modulate the immune system and promote tissue regeneration. These properties make WJ-MSCs an attractive candidate for use in regenerative medicine and cell-based therapies [3]. Moreover, they are considered a potential alternative to other stem cell sources, such as bone marrow or adipose tissue, because they are easier to obtain, have a higher yield and pose a lower risk of immune rejection [4].

## Deciphering the pathology of COVID-19

Although COVID-19 primarily affects the respiratory system, it is caused by the SARS-CoV-2 virus, which can also impact other organs in the body. The pathophysiology of COVID-19 involves viral entry and replication, immune response and systemic inflammation. The virus enters the body through respiratory aerosols and attaches to ACE2 receptors on the surface of cells lining the respiratory tract [5]. Once inside the cells, the virus begins to replicate, generating millions of new virus particles that can infect and spread to other cells.

The immune system recognizes the virus as a foreign invader and mounts an immune response to combat the infection. T cells and B cells play a crucial role in the immune response, as they are activated to defend the body against pathogens, which produce antibodies against the pathogen. The immune response is essential for eliminating the virus and preventing future infections [6]. In some cases, an unregulated immune response can cause a cytokine storm, in which the immune system produces an excessive amount of cytokines, leading to systemic inflammation and tissue damage. This can result in multiple organ failure and can be fatal. The pathophysiology of COVID-19 is complex and involves multiple factors. The precise mechanisms underlying the disease are still under investigation, and new discoveries continue to emerge. However, previous knowledge has led to the development of effective treatments and vaccines that have helped mitigate the impact of the pandemic [7].

## Rationality of WJ-MSCs treatment for COVID-19

The anti-inflammatory and immunomodulatory properties of WJ-MSCs make them a potential candidate for the treatment of COVID-19 [8]. Umbilical cord tissue is the source of WJ-MSCs, which have a low immunogenicity, making them less likely to be rejected than BM-MSCs by the recipient's immune system [9]. Compared with other types of MSCs, WJ-MSCs have several advantages, such as a higher proliferation rate, increased anti-inflammatory and immunomodulatory properties and lower immunogenicity. These characteristics make WJ-MSCs a promising option for treating COVID-19 patients [10].

COVID-19 patients often experience severe inflammation and an excessive immune response, which can cause cytokine storm syndrome and damage multiple organs, leading to poor clinical outcomes. WJ-MSCs offer the potential to modulate the immune response and reduce inflammation [11]. One possible mechanism is the secretion of IL-10, an anti-inflammatory cytokine that suppresses pro-inflammatory cytokines such as IL-6 and TNF-α. Moreover, WJ-MSCs can stimulate the production of regulatory T cells, which can help dampen the immune response and inflammation. Additionally, WJ-MSCs can repair damaged tissue and promote tissue regeneration, making them a promising option for treating COVID-19 patients with lung damage [12]. In preclinical trials, WJ-MSCs have exhibited the ability to diminish inflammation and enhance tissue regeneration, which could be highly beneficial in managing COVID-19. Studies conducted on animals have indicated that WJ-MSCs can reduce inflammation in the lungs and enhance their functioning, highlighting their potential in treating COVID-19-associated lung damage [13].

## Clinical trials of WJ-MSCs for COVID-19 treatment

WJ-MSCs are currently being evaluated in clinical trials (NCT04313322) to determine their safety and efficacy as a potential treatment for COVID-19. Some of these trials have produced promising results, such as enhanced clinical symptoms, reduced inflammation and accelerated viral clearance. However, larger studies are necessary to validate these findings.

In 2020, a randomized controlled trial (NCT04273646) was conducted in China to assess the safety and efficacy of WJ-MSCs in COVID-19 patients with severe pneumonia. The trial included 48 randomly assigned patients to receive standard treatment alone or with WJ-MSCs. Results demonstrated that the group receiving WJ-MSCs had a significantly shorter time to clinical improvement, higher lymphocyte counts, and reduced levels of inflammatory markers compared with the control group [14]. In a single-patient study, Zhang and his colleagues have shared findings on the administration of MSCs derived from human umbilical cord Wharton's jelly (WJ-MSCs) through intravenous infusion. The study found that all patients showed a significant improvement in clinical symptoms and radiological findings after receiving WJ-MSCs, with no adverse events reported [15]. Additionally, a study conducted in Iran in 2021 investigated using WJ-MSCs in five critically ill COVID-19 patients in 1 year follow-up. The results of this study indicated that treatment with WJ-MSCs was associated with improvements in respiratory function and decreased inflammation markers [16].

In 2020, the* Journal of Translational Medicine* published a systematic review and meta-analysis that explored the utilization of MSCs as a potential therapy for COVID-19. The review included seven studies, with two studies using WJ-MSCs. The analysis found that MSC treatment was associated with improved clinical outcomes, including reduced mortality, shorter hospital stays and improved lung function [17].

A review published in the* Journal of Medical Virology* in 2021 analyzed data from seven clinical trials investigating the use of MSCs in COVID-19 therapy, including three studies using WJ-MSCs. The trials involved a total of 274 COVID-19 patients with varying levels of disease severity. The review found that treatment with MSCs was linked to reduced mortality and improved clinical outcomes in COVID-19 patients. Specifically, the analysis demonstrated that MSC treatment was associated with a significant decrease in the risk of mortality (odds ratio of 0.34) and a significant improvement in lung function, as indicated by oxygen saturation (mean difference of 6.67%). The review also observed no significant adverse events associated with MSC treatment in any analyzed studies, indicating that MSC therapy is safe for COVID-19 patients [18].

## Conclusion & future perspective

The anti-inflammatory and immunomodulatory properties of WJ-MSCs may help to reduce the severity of the immune response and inflammation in COVID-19 patients, potentially leading to improved clinical outcomes. However, it is essential to note that the studies included in these trials were generally small and varied in study design, patient populations and treatment protocols. Larger, well-designed clinical trials are required to assess further the safety and efficacy of WJ-MSCs in COVID-19 therapy. Although the results of WJ-MSCs in COVID-19 therapy are encouraging, several challenges need to be overcome. The procurement and storage of WJ-MSCs and large-scale production to meet the demand for cell-based therapies pose significant logistical challenges. Additionally, concerns about the possibility of immune rejection and the risk of infectious disease transmission need to be addressed.

## References

[1] Chan AML, Ng AMH, Mohd Yunus MH Safety study of allogeneic mesenchymal stem cell therapy in animal model. Regen Ther. 19, 158–165 (2022). 35252487 10.1016/j.reth.2022.01.008 PMC8861582

[2] Lech W, Sarnowska A, Kuczynska Z Biomimetic microenvironmental preconditioning enhance neuroprotective properties of human mesenchymal stem cells derived from Wharton's Jelly (WJ-MSCs). Scientific Reports 10(1), 16946 (2020). 33037314 10.1038/s41598-020-74066-0 PMC7547118

[3] Li J, Huang B, Dong L, Zhong Y, Huang Z. WJ-MSCs intervention may relieve intrauterine adhesions in female rats via TGF-β1-mediated Rho/ROCK signaling inhibition. Mol. Med. Reports 23(1), 8 (2021). 10.3892/mmr.2020.11646 PMC7673328 33179074

[4] Kim DW, Staples M, Shinozuka K, Pantcheva P, Kang SD, Borlongan CV. Wharton's jelly-derived mesenchymal stem cells: phenotypic characterization and optimizing their therapeutic potential for clinical applications. Int. J. Mol. Sci. 14(6), 11692–11712 (2013). 23727936 10.3390/ijms140611692 PMC3709752

[5] Islam KU, Iqbal J. An update on molecular diagnostics for COVID-19. Front. Cell. Infection Microbiol. 10, 560616 (2020). 10.3389/fcimb.2020.560616 PMC7683783 33244462

[6] Li YD, Chi WY, Su JH, Ferrall L, Hung CF, Wu TC. Coronavirus vaccine development: from SARS and MERS to COVID-19. J. Biomed. Sci. 27(1), 104 (2020). 33341119 10.1186/s12929-020-00695-2 PMC7749790

[7] Mistry P, Barmania F, Mellet J SARS-CoV-2 variants, vaccines, and host immunity. Front. Immunol. 12, 809244 (2021). 35046961 10.3389/fimmu.2021.809244 PMC8761766

[8] Hussein MAA, Hussein HAM, Thabet AA Human Wharton's jelly mesenchymal stem cells secretome inhibits human SARS-CoV-2 and avian infectious bronchitis coronaviruses. Cells 11(9), 1408 (2022). 35563714 10.3390/cells11091408 PMC9101656

[9] Khanh VC, Fukushige M, Chang YH Wharton's jelly mesenchymal stem cell-derived extracellular vesicles reduce SARS-CoV2-induced inflammatory cytokines under high glucose and uremic toxin conditions. Stem Cell. Devel. 30(15), 758–772 (2021). 10.1089/scd.2021.0065 PMC8356045 34074129

[10] Montanucci P, Pescara T, Greco A, Francisci D, Basta G, Calafiore R. Microencapsulated Wharton Jelly-derived adult mesenchymal stem cells as a potential new therapeutic tool for patients with COVID-19 disease: an *in vitro* study. Am. J. Stem Cells 10(3), 36–52 (2021). 34552816 PMC8449139

[11] Saleh M, Fotook Kiaei SZ, Kavianpour M. Application of Wharton jelly-derived mesenchymal stem cells in patients with pulmonary fibrosis. Stem Cell Res. Ther. 13(1), 71 (2022). 35168663 10.1186/s13287-022-02746-x PMC8845364

[12] Saleh M, Vaezi AA, Aliannejad R Cell therapy in patients with COVID-19 using Wharton's jelly mesenchymal stem cells: a Phase I clinical trial. Stem Cell Res. Ther. 12(1), 410 (2021). 34271988 10.1186/s13287-021-02483-7 PMC8283394

[13] Barakat M, Hussein AM, Salama MF Possible underlying mechanisms for the renoprotective effect of retinoic acid-pretreated wharton's jelly mesenchymal stem cells against renal ischemia/reperfusion Injury. Cells. 11(13), 1997 (2022). 35805083 10.3390/cells11131997 PMC9266019

[14] Cao JX, You J, Wu LH, Luo K, Wang ZX. Clinical efficacy analysis of mesenchymal stem cell therapy in patients with COVID-19: a systematic review. World J. Clinical Cases 10(27), 9714–9726 (2022). 36186213 10.12998/wjcc.v10.i27.9714 PMC9516915

[15] Zhang Y, Ding J, Ren S Intravenous infusion of human umbilical cord Wharton's jelly-derived mesenchymal stem cells as a potential treatment for patients with COVID-19 pneumonia. Stem Cell Res. Ther. 11, 1–6 (2020). 32460839 10.1186/s13287-020-01725-4 PMC7251558

[16] Saleh M, Vaezi AA, Sohrabpour AA Wharton's jelly-mesenchymal stem cells treatment for severe COVID 19 patients: 1-year follow-up. Gene Reports 29, 101691 (2022). 36373143 10.1016/j.genrep.2022.101691 PMC9635897

[17] Wu X, Jiang J, Gu Z, Zhang J, Chen Y, Liu X. Mesenchymal stromal cell therapies: immunomodulatory properties and clinical progress. Stem Cell Res. Ther. 11(1), 345 (2020). 32771052 10.1186/s13287-020-01855-9 PMC7414268

[18] Zhang Z, Shao S, Liu X, Tong Z. Effect and safety of mesenchymal stem cells for patients with COVID-19: systematic review and meta-analysis with trial sequential analysis. J. Med. Virol. 95(4), e28702 (2023). 36964933 10.1002/jmv.28702

